# iTRAQ-based comparative proteomics reveal an enhancing role of PRDX6 in the freezability of Mediterranean buffalo sperm

**DOI:** 10.1186/s12864-023-09329-x

**Published:** 2023-05-05

**Authors:** Xi Luo, Mingming Liang, Shihai Huang, Qingsong Xue, Xuan Ren, Yanfang Li, Jinli Wang, Deshun Shi, Xiangping Li

**Affiliations:** 1 State Key Laboratory for Conservation and Utilization of Subtropical Agro-Bioresources, Guangxi Key Laboratory of Animal Breeding and Disease Control, Guangxi University, Nanning, 530005 China; 2 Liuzhou Maternity and Child Healthcare Hospital, Liuzhou, 545001 Guangxi China; 3 College of life science and technology, Guangxi University, Nanning, China

**Keywords:** Sperm freezability, PRDX6, Cryopreservation, iTRAQ-based proteomics, Mediterranean buffalo

## Abstract

**Background:**

Semen cryopreservation is a critical tool for breed improvement and preservation of biodiversity. However, instability of sperm freezability affects its application. The Mediterranean buffalo is one of the river-type buffaloes with the capacity for high milk production. Until now, there is no specific cryopreservation system for Mediterranean buffalo, which influences the promotion of excellent cultivars. To improve the semen freezing extender used in cryopreservation of Mediterranean buffalo, different protein datasets relating to freezability sperm were analyzed by iTRAQ-based proteomics. This study will be beneficial for further understanding the sperm freezability mechanism and developing new cryopreservation strategy for buffalo semen.

**Results:**

2652 quantified proteins were identified, including 248 significantly differentially expressed proteins (DEP). Gene Ontology (GO) analysis indicated that many these were mitochondrial proteins, enriched in the molecular function of phospholipase A2 activity and enzyme binding, and biological processes of regulation of protein kinase A signaling and motile cilium assembly. Kyoto Encyclopedia of Genes and Genomes (KEGG) pathway enrichment analysis identified 17 significant pathways, including oxidative phosphorylation (OXPHOS). Furthermore, 7 DEPs were verified using parallel reaction monitoring or western blot, which confirmed the accuracy of the iTRAQ data. Peroxiredoxin 6 (PRDX6), which expressed 1.72-fold higher in good freezability ejaculate (GFE) compared to poor freezability ejaculate (PFE) sperms, was selected to explore the function in sperm freezability by adding recombinant PRDX6 protein into the semen freezing extender. The results showed that the motility, mitochondrial function and in vitro fertilization capacity of frozen-thawed sperm were significantly increased, while the oxidation level was significantly decreased when 0.1 mg/L PRDX6 was added compared with blank control.

**Conclusions:**

Above results revealed the metabolic pattern of freezability of Mediterranean buffalo sperms was negatively associated with OXPHOS, and PRDX6 had protective effect on cryo-damage of frozen-thawed sperms.

**Supplementary Information:**

The online version contains supplementary material available at 10.1186/s12864-023-09329-x.

## Introduction

Sperm cryopreservation is an important tool for breed improvement and preservation of genetic resources. The freeze-thaw procedure inevitably accompanies cryo-damage, which is caused by extreme osmotic change, cold shock, intracellular ice crystal formation, excessive generation of reactive oxygen species (ROS) and a change in the antioxidant defense system [1, 2]. These processes eventually lead to disruption of sperm morphology and physiological function. In order to predict and improve cryopreservation outcome, several candidate proteins have been found for freezability biomarkers in some laboratories.

The solute carrier family 2 member 3, heat shock protein 90-KDa alpha A1 and copper- and zinc-containing superoxide dismutase are identified as boar freezability biomarkers by western blot (WB) [3]. Enolase1 and glucose-6-phosphate isomerase are identified as human freezability biomarkers by WB and immunofluorescence (IF) [4]. In addition, high throughput technologies such as sodium dodecyl sulfate-polyacrylamide gel electrophoresis (SDS-PAGE) and protein microarrays have been used to screen the freezabilty biomarkers. Acrosin binding protein, triosephosphate isomerase [5], voltage-dependent anion channel 2 [6] and pSer levels in HSP70 [7] are thought to be good predictors for freezability property of boar sperm. Several proteins including glutathione s-transferase mu 5, voltage-dependent anion-selective channel protein 2 and ATP synthase subunit beta are shown to be useful markers for selecting high cryotolerance of bull epididymal spermatozoa [8]. These studies have shown that it is feasible to screen potential sperm freezability biomarkers. However, the methods are confined to some protein analyses and 2-dimensional SDS-PAGE technology.

Compared with 2-dimensional SDS-PAGE and label free quantification, isobaric tags for relative and absolute quantitation (iTRAQ) have the advantages of robustness to variations in sensitivity and chromatographic reproducibility [9]. iTRAQ-coupled LC-MS/MS technology is currently being used to track various biomarkers in spermatogenesis [10, 11], sperm maturation [12], concentration and motility changes of ejaculates [13], cryoinjury [14, 15] and fertility [16, 17]. The parallel reaction monitoring (PRM) technology is recently developed in the use of targeted mass spectrometry. The precision, resolution and sensitivity of this method have significant improvement when compared to either selected reaction monitoring or WB [15]. Recently, PRM has been used for the identification and quantification of target proteins, which was also validated for proteomics data [15].

Mediterranean buffaloes produce milk of high nutritional value. However, the instability of sperm cryopreservation has influenced the promotion of excellent cultivars. In order to improve the semen freezing extender used in cryopreservation, different protein datasets relating to freezability sperm were analyzed by iTRAQ-based proteomics and these were validated either by PRM or WB. PRDX6 was selected as the freezability candidate protein and added to semen freezing extender for validation. Subsequently, the motility, antioxidant and oxidation levels, mitochondrial membrane potential (MMP), sperm capacitation and acrosome reaction of frozen-thawed sperms were determined. This study enriched the theoretical basis for the sperm freezability mechanism, in addition, it demonstrated the feasibility of adding candidate protein to semen freezing extender to obtain the optimum quality sperms after cryopreservation for Mediterranean buffalo.

## Results

### Freezability and quality classification of buffalo sperms

According to the motility of post-thaw sperms (≥ 30% or < 30%), three bulls with the motility of 31.3%, 31.3% and 35.8% were defined as GFE; another three bulls with the motility of 10.0%, 18.0% and 18.3% were grouped as PFE. As shown in Table 1, all the kinetic and conventional semen parameters of fresh semen were not significantly different between the GFE and PFE groups. However, the motility of frozen-thawed sperms in the GFE group were higher than PFE group (*P <* 0.05).

**Table 1:** The kinetic and quality parameters of Mediterranean buffalo sperm in the GFE and PFE groups

Parameter	Freezability	
	GFE	PFE
Ejaculate volume (mL)	6.68 ± 0.99	6.98 ± 0.67
Concentration(10^8^/mL)	14.84 ± 1.35	14.97 ± 1.52
Total motility (TM, %)	67.80 ± 0.60	67.40 ± 0.80
Curvilinear velocity (VCL; µm/s)	126.41 ± 7.15	137.86 ± 6.96
Straight-line velocity (VSL; µm/s)	35.39 ± 2.59	35.76 ± 3.38
Average path velocity (VAP; µm/s)	68.42 ± 4.06	70.59 ± 4.88
Linearity (LIN; %)	27.00 ± 1.00	25.00 ± 1.00
Straightness (STR; %)	48.00 ± 1.00	46.00 ± 1.00
Amplitude of lateral head displacement (ALH; µm)	3.46 ± 0.19	3.83 ± 0.10
Beat cross-frequency (BCF; Hz)	10.28 ± 0.42	9.41 ± 0.33
Thawed sperm total motility (TM, %)	33.00 ± 1.00 ^a^	17.00 ± 3.00^b^

Note: The values (mean ± SEM) having different superscripts differ significantly (*P* < 0.05) for a given parameter. GFE, good freezability ejaculate; PFE, poor freezability ejaculate

### Identification of protein files in GFE and PFE sperm

**Fig. 1:**
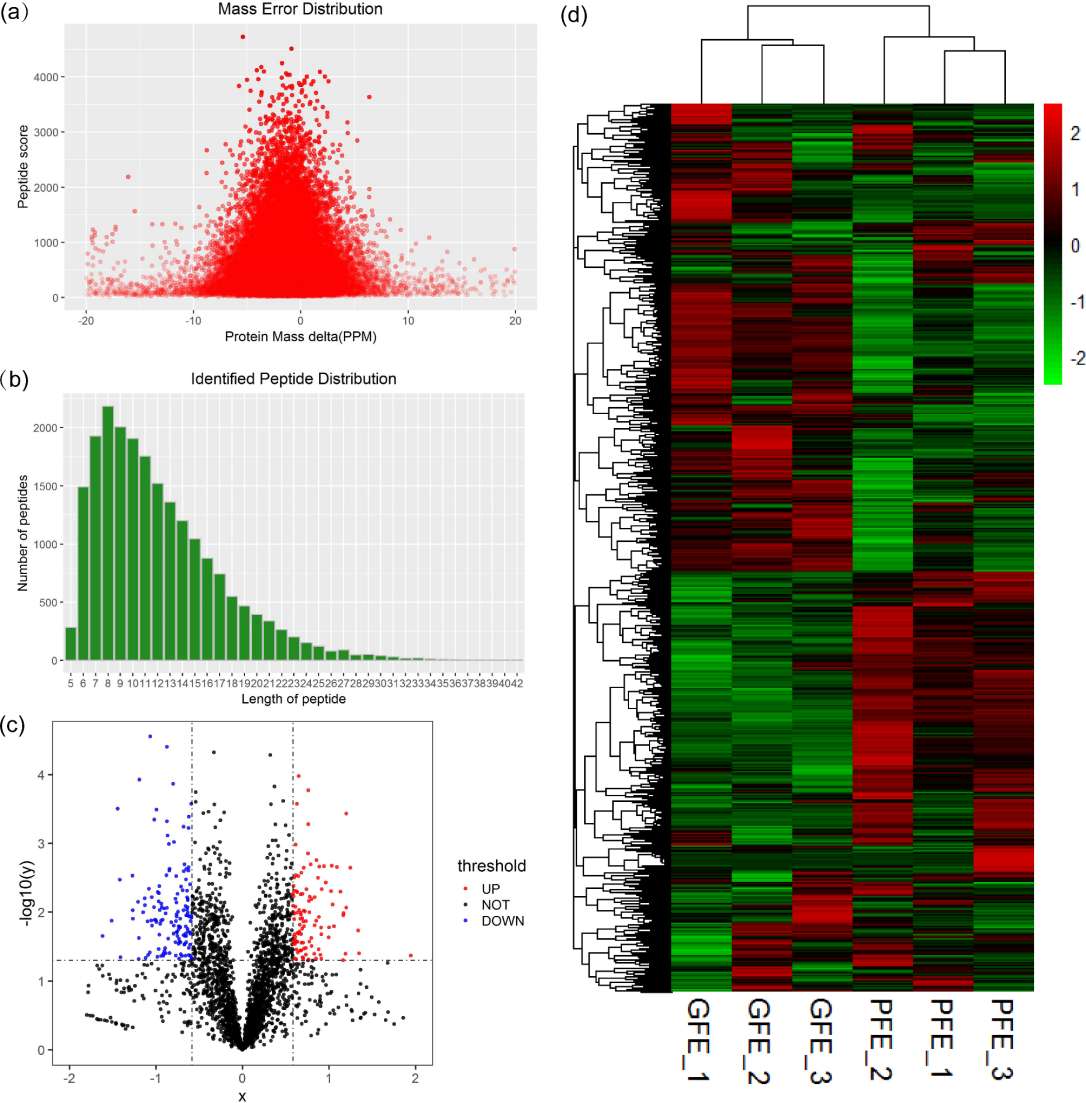
Analysis of the quality control and cluster analysis of the differentially regulated proteins (**a**) The mass error distribution. (**b**) The peptide length distribution. (**c**) The volcano plot of GFE vs. PFE. (**d**) The heatmap of differentially regulated proteins based on hierarchical cluster analysis. The protein abundance profiles with selective features of the GFE (GFE1, GFE2 and GFE3) and PFE (PFE1, PFE2 and PFE3) groups were calculated in the context of all 248 differentially regulated proteins after normalization using hierarchical cluster analysis. The red and green colors represented upregulated and downregulated proteins, respectively

The distribution of the mass error was near zero and most of the identified peptides were within 10 ppm (Fig. 1a). The lengths of most peptides were distributed within 6 to 20 amino-acid residues, which were in agreement with their properties, and thus sample preparation met the technically standard (Fig. 1b). Hierarchical clustering analysis of proteome variation revealed two statistically robust clades (Fig. 1c).

3730 proteins were identified in total and 2652 proteins were quantified in the two groups. On the basis of FC > 1.5 or < 0.67 and p-value < 0.05, compared with PFE group, 109 proteins were significantly upregulated in the GFE group and 139 proteins were significantly downregulated (Fig. 1d). The top 10 of the upregulated and downregulated proteins were showed in Tables 2 and 3, respectively.

**Table 2:** The top 10 upregulated proteins between the GFE vs. PFE groups

Protein ID	Protein name	Fold change	p-value
P35722	Neurogranin	4.289980732	0.017412649
F1MFZ5	Unc-45 myosin chaperone B	3.852272727	0.042570126
F1N2G1	Biorientation of chromosomes in cell division 1 like 1	2.545283019	0.039614258
Q2NKS8	Peptidylprolyl isomerase	2.530956848	0.018492024
Q58DH8	Flap endonuclease 1	2.377313433	0.002265361
P80177	Macrophage migration inhibitory factor	2.298326601	0.008590409
E1BMH3	Chromosome 17 C4orf33 homolog	2.297549592	0.000368929
E1BE86	Copper chaperone for superoxide dismutase	2.284143519	0.040178377
A0A3Q1N762	ATR serine/threonine kinase	2.248561565	0.010308612
A0A3Q1LY87	ENAH actin regulator	2.244583808	0.011062205

**Table 3:** The top 10 downregulated proteins between the GFE vs. PFE groups

Protein ID	Protein name	Fold change	p-value
Q95142	Retinal rod rhodopsin-sensitive cGMP 3’,5’-cyclic phosphodiesterase subunit delta	0.239761727	0.030535073
G3N1S7	Uncharacterized protein	0.325710754	0.022237397
A0A3Q1N448	GTP binding protein overexpressed in skeletal muscle	0.35016835	0.013305188
E1BD64	Mitochondrial pyruvate carrier	0.367672664	0.000314321
E1BJC4	Piwi like RNA-mediated gene silencing 1	0.374100719	0.003379927
E1BP97	Protein disulfide isomerase like, testis expressed	0.376311337	0.04525808
P46195	Guanylate kinase	0.414563107	0.002952354
Q24JZ0	NADH dehydrogenase (Ubiquinone) 1 beta subcomplex, 1, 7 kDa	0.414846188	0.009368009
Q58D62	Fetuin-B	0.41581063	0.012465133
A0A3Q1M728	Uncharacterized protein	0.433363554	0.007084361

### Functional enrichment analysis of proteins

Functional enrichment analyses based on GO, KEGG pathway and COG were carried out to reveal the characteristics of the GFE and PFE DEPs.

**Fig. 2:**
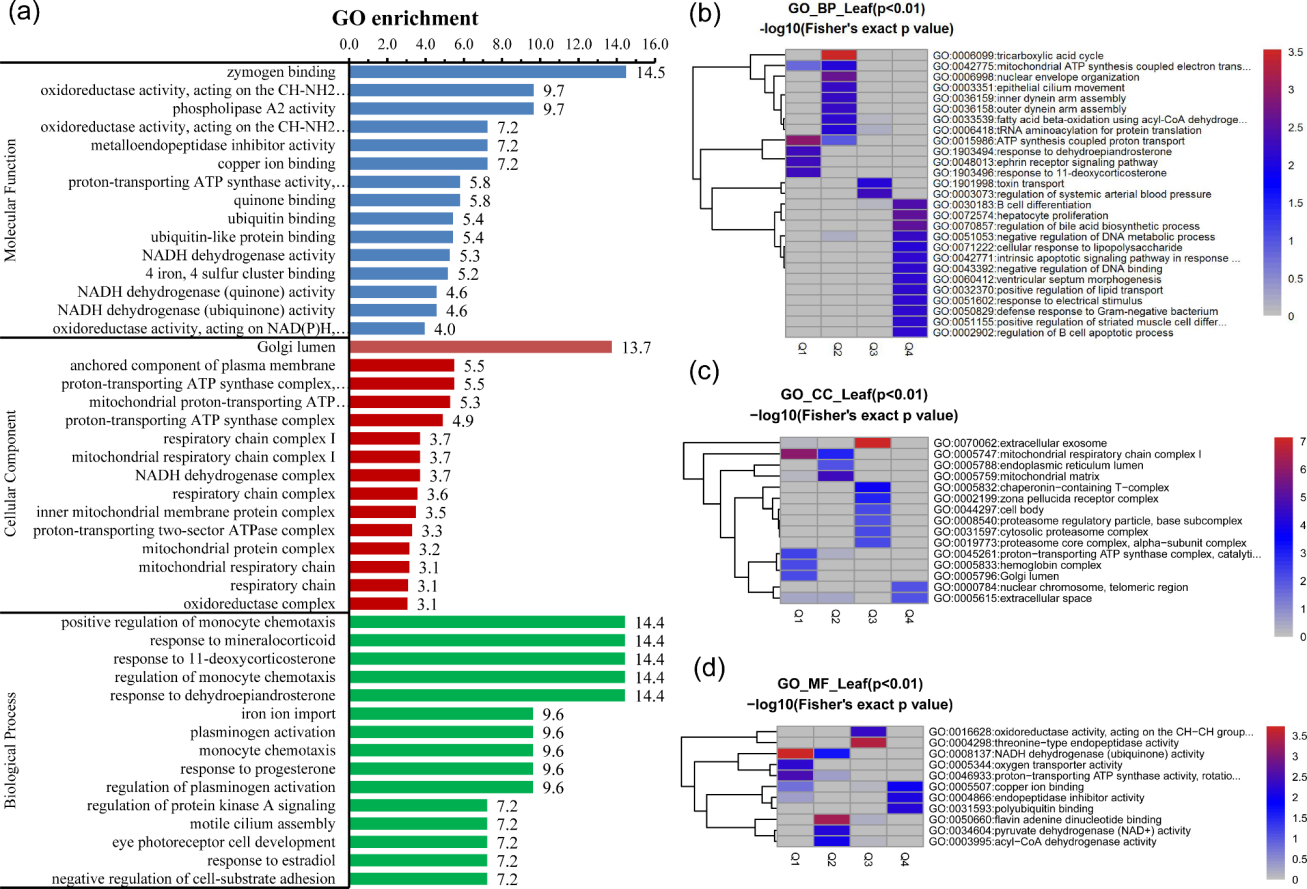
Bioinformatics analysis of the GFE vs. PFE groups (**a**) Gene ontology annotation of the differentially regulated proteins based on cellular component, biological process and molecular function (GFE vs. PFE groups). The number above the bars represented the enrichment factor. (**b**) Biological process-based clustering analysis. (**c**) Cellular component-based clustering analysis. (**d**) Molecular function-based clustering analysis. DEPs were divided into Q1 (< 0.67), Q2 (0.67-1), Q3 (1-1.5) and Q4 (> 1.5) according to fold changes (GFE vs. PFE). The redder the color of the blocks, the more significant the enrichment. The greyer the color of the blocks, the lower the significant of enrichment

Based on the GO database, the top GO terms enriched from the dysregulated proteins included molecular function of zymogen binding, phospholipase A2 activity, NADH dehydrogenase activity, cellular component of Golgi lumen, anchored component of plasma membrane, respiratory chain complex, the biological processes of response to dehydroepiandrosterone, regulation of protein kinase A signaling and iron ion import (Fig. 2a).

To further examine the similarities and differences in the signaling pathway of DEPs, these were divided into Q1 (< 0.67), Q2 (0.67-1), Q3 (1-1.5) and Q4 (> 1.5) according to fold change (GFE vs. PFE). The most enriched biological processes of Q1, Q2, Q3 and Q4 proteins were ATP synthesis coupled proton transport, tricarboxylic acid cycle, regulation of systemic arterial blood pressure and hepatocyte proliferation, respectively (Fig. 2b). The top enriched cellular components of Q1, Q2, Q3 and Q4 DEPs were the mitochondrial respiratory chain complex, mitochondrial matrix, extracellular exosome and extracellular space, respectively (Fig. 2c). For molecular function analysis, the Q1, Q2, Q3 and Q4 DEPs showed predominant enrichment in NADH dehydrogenase (ubiquinone), pyruvate dehydrogenase (NAD+) and threonine-type endopeptidase activities as well as copper ion binding, respectively (Fig. 2d).

**Fig. 3:**
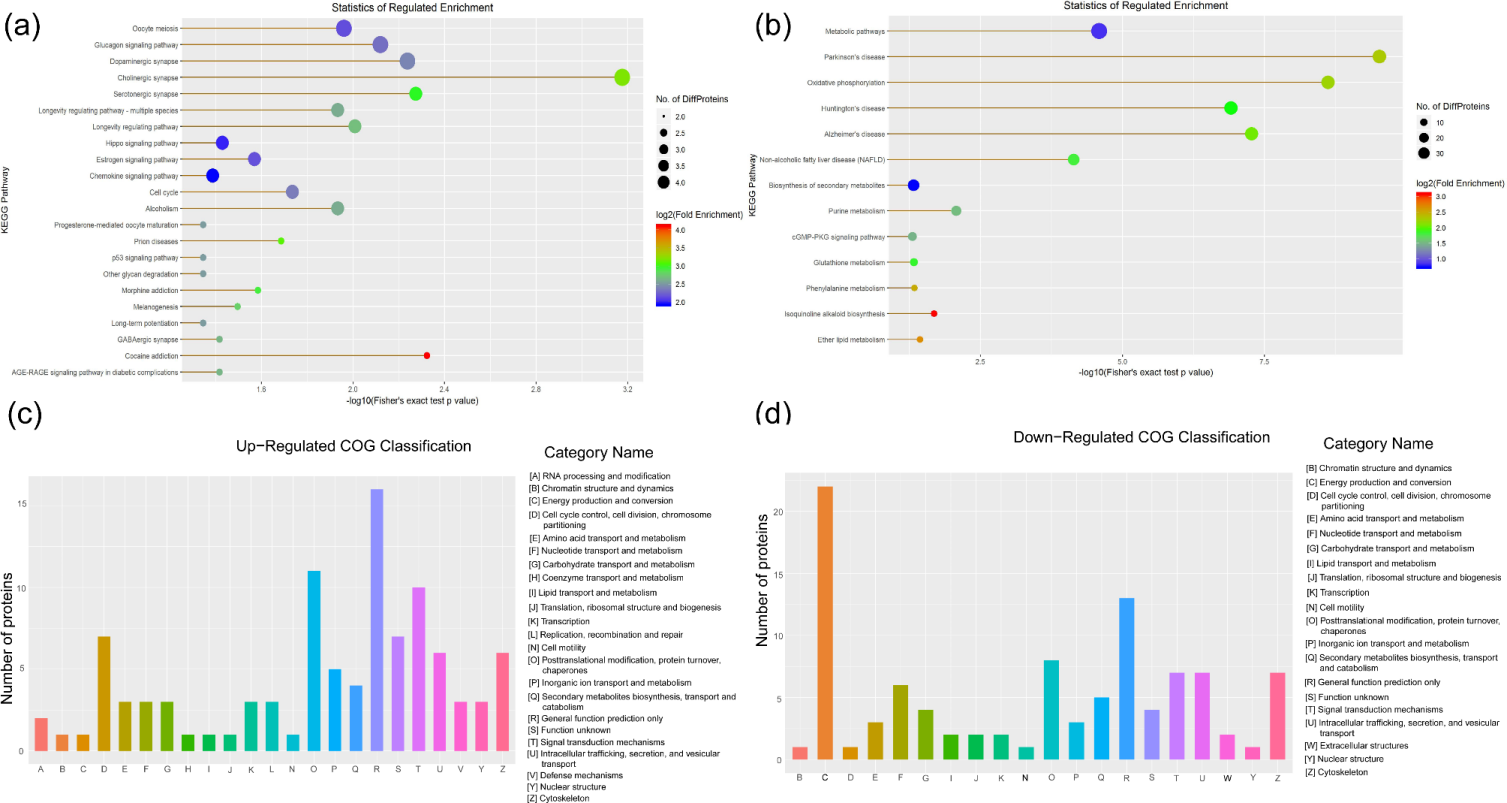
Bioinformatics analysis of the GFE vs. PFE groups (**a**) KEGG pathway enrichment analysis of the upregulated proteins (GFE vs. PFE). (**b**) KEGG pathway enrichment analysis of the downregulated proteins (GFE vs. PFE). The size of the circle represented the number of proteins, and the color represented the p value. (**c**) Distribution of COG/KOG functional classes of the upregulated proteins (GFE vs. PFE). (**d**) Distribution of COG/KOG functional classes of the downregulated proteins (GFE vs. PFE).

By using KEGG pathway enrichment analysis, this study identified that the upregulated proteins were involved in estrogen, Hippo and p53 signaling pathways as well as the cell cycle (Fig. 3a). The downregulated proteins were involved in OXPHOS, purine metabolism, phenylalanine metabolism and glutathione metabolism (Fig. 3b). Among these, cAMP-dependent protein kinase catalytic subunit beta (PRKACB) and cyclic AMP-responsive element-binding protein 5 (CREB5) acted as key nodes with outstanding performance. They appeared frequently in most of upregulated pathways and were closely linked to them.

There were 202 proteins annotated against the COG/KOG database. In all the functional ontologies, the R (general function prediction only), O (posttranslational modification, protein turnover, chaperones) and T groups (signal transduction mechanisms) had the most upregulated proteins (Fig. 3c), while the C (energy production and conversion) and R groups (general function prediction only) had the most downregulated proteins (Fig. 3d). This indicated that these differentially abundant proteins played crucial roles in energy production and conversion and general function prediction in eukaryotes.

### Protein–protein interaction (PPI) network construction

**Fig. 4:**
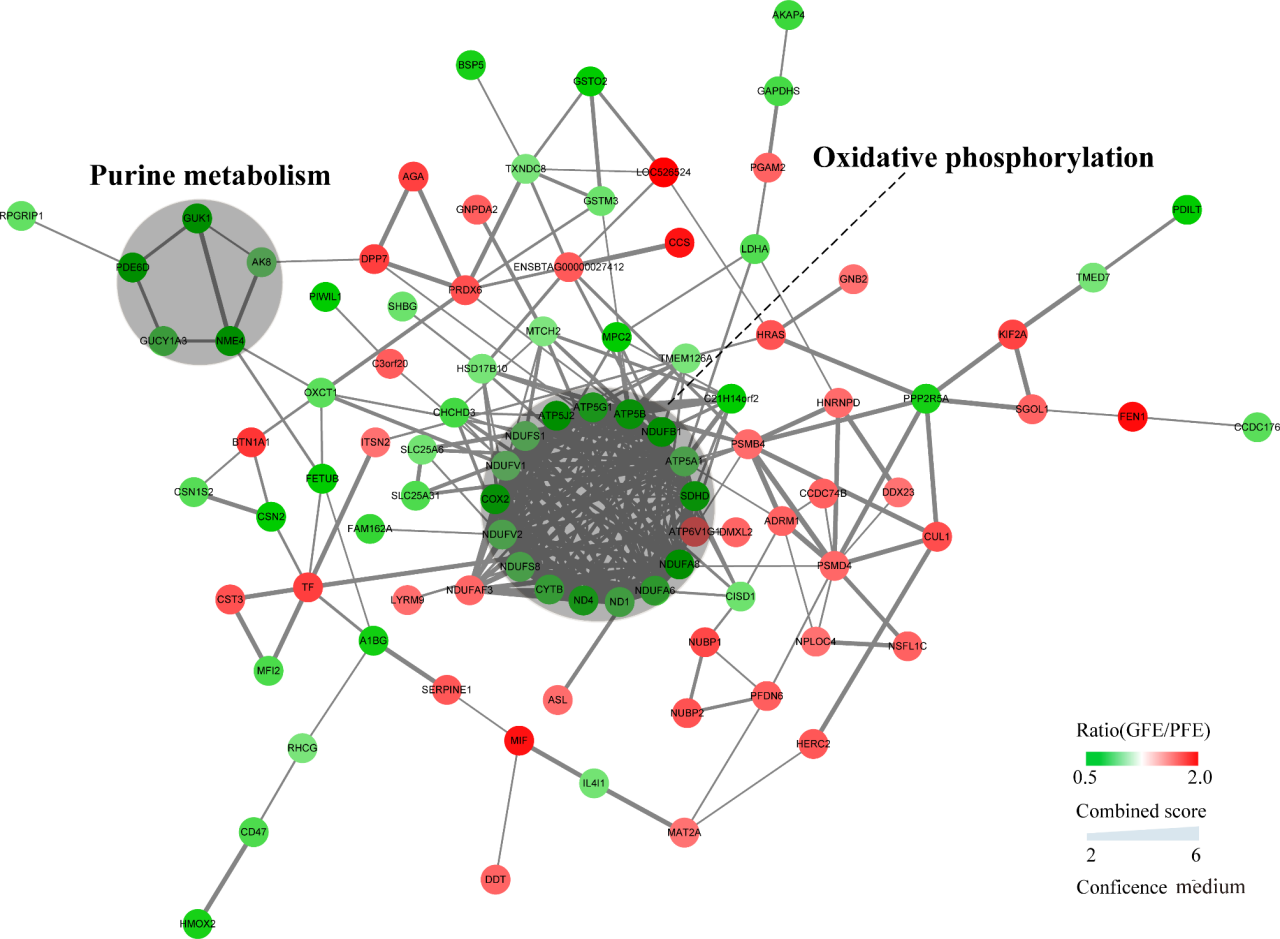
PPI network analysis of the differentially regulated proteins (GFE vs. PFE groups) The circle nodes represented the differentially expressed proteins and the lines represented the interactions between the two proteins. The red and green colors represented the upregulated and downregulated proteins

When comparing the GFE vs. PFE groups, there were two clusters enriched. Sixteen decreasing proteins were clustered in OXPHOS, and most of them were mitochondrial complex proteins as well mitochondrial dehydrogenase. Five reduced proteins were clustered in purine metabolism (Fig. 4).

### Validation of selected proteins by PRM or WB

**Fig. 5:**
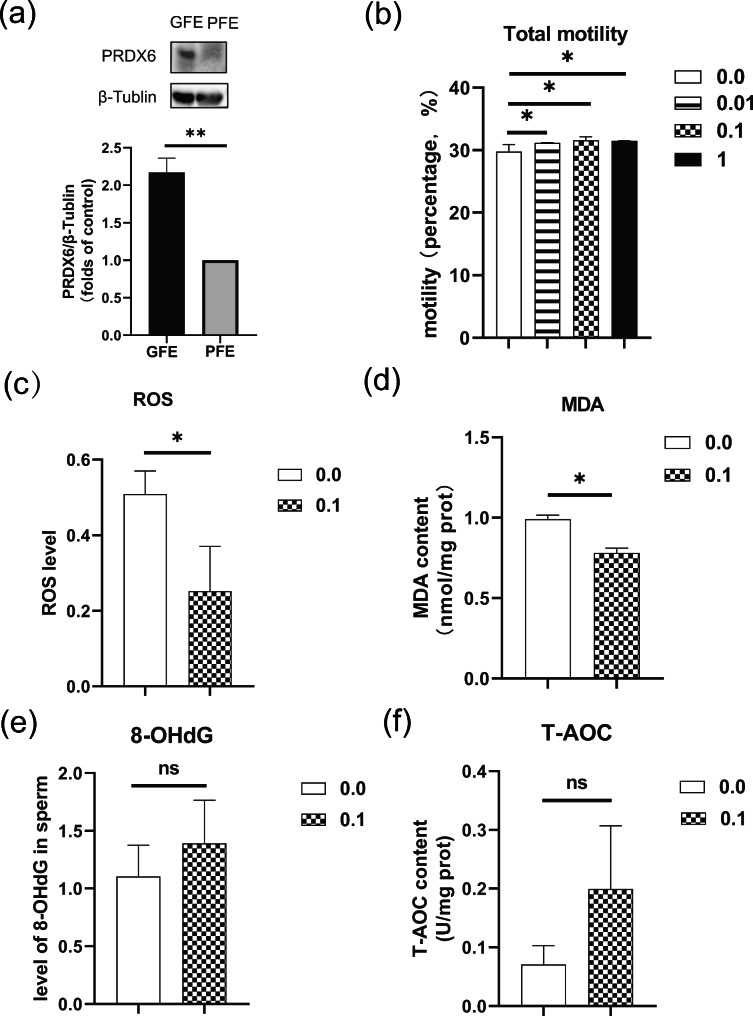
(**a**) Western blot analysis of PRDX6 abundance in GFE and PFE sperms. The samples derived from the same experiment and all blots were processed in parallel. The bars represented the mean values ± SEM of the intensity of all the bands per lane expressed in arbitrary units (A.U.) using sperm in three independent experiments. (**b**) The effect of PRDX6 (0.0, 0.01, 0.1 and 1 mg/L) on the frozen-thawed sperm motility of Mediterranean buffaloes. (**c**) The effect of PRDX6 on the ROS levels of frozen-thawed sperm. (**d**) The effect of PRDX6 on the DNA oxidation of frozen-thawed sperm. (**e**) The effect of PRDX6 on the lipid oxidation of frozen-thawed sperm. (**f**) The effect of PRDX6 on the total antioxidant capacity of frozen-thawed sperm. The t test was used to compare data between two groups and variance (ANOVA) was used to compare data between multiple groups. *P < 0.05, **P < 0.01

To verify the accuracy of the proteomics data, six DEPs were quantified by PRM (Table 4) and PRDX6 was quantified by WB (Fig. 5a). All the validation results showed the same trend in PRM and WB experiments when compared with the results of iTRAQ, which indicated that the iTRAQ results were credible and could be used for further analysis.

**Table 4:** Confirmation of 6 DEPs as detected during iTRAQ analysis by using PRM analysis

Protein ID	Description (NCBI)		Fold change (iTRAQ)	Fold change (PRM)
P15690		NADH-ubiquinone oxidoreductase 75 kDa subunit, mitochondrial	0.649384656	0.749665923
Q2KJE5	Glyceraldehyde-3-phosphate dehydrogenase, testis-specific		0.577890724	0.722641837
E1BD64	Mitochondrial pyruvate carrier		0.367672664	0.684912461
F1MYH5	A-kinase anchoring protein 4		0.56593542	0.632369262
F1N2F2	Phosphoglycerate mutase		1.590575011	1.899681405
Q24JZ7	Succinyl-CoA:3-ketoacid-coenzyme A transferase		0.608101513	0.603642301

### The effects of PRDX6 on oxidative and antioxidative levels of frozen-thawed sperm

Compared with the blank control (29.80 ± 1.09%), the motility of frozen-thawed sperms was increased when 0.01, 0.1 and 1 mg/L PRDX6 was added (31.20 ± 0.45%, 31.60 ± 0.55 and 31.50 ± 1.00, respectively) (*P* < 0.05) (Fig. 5b). Among all the treatment groups, sperms in the 0.1 mg/L PRDX6 group showed the highest mobility. Therefore, this concentration of PRDX6 was selected for use in subsequent experiments.

Compared with the blank control, the ROS and MDA levels in the 0.1 mg/L PRDX6 group were decreased (*P* < 0.05) (Fig. 5c, d), while 8-OHdG level showed only a slight decreased (*P* > 0.05) (Fig. 5e). The T-AOC level in the 0.1 mg/L PRDX6 group was slightly higher than blank control (*P* > 0.05) (Fig. 5f).

### Effect of PRDX6 on fertilization capability and mitochondrial function of frozen-thawed sperm

**Fig. 6:**
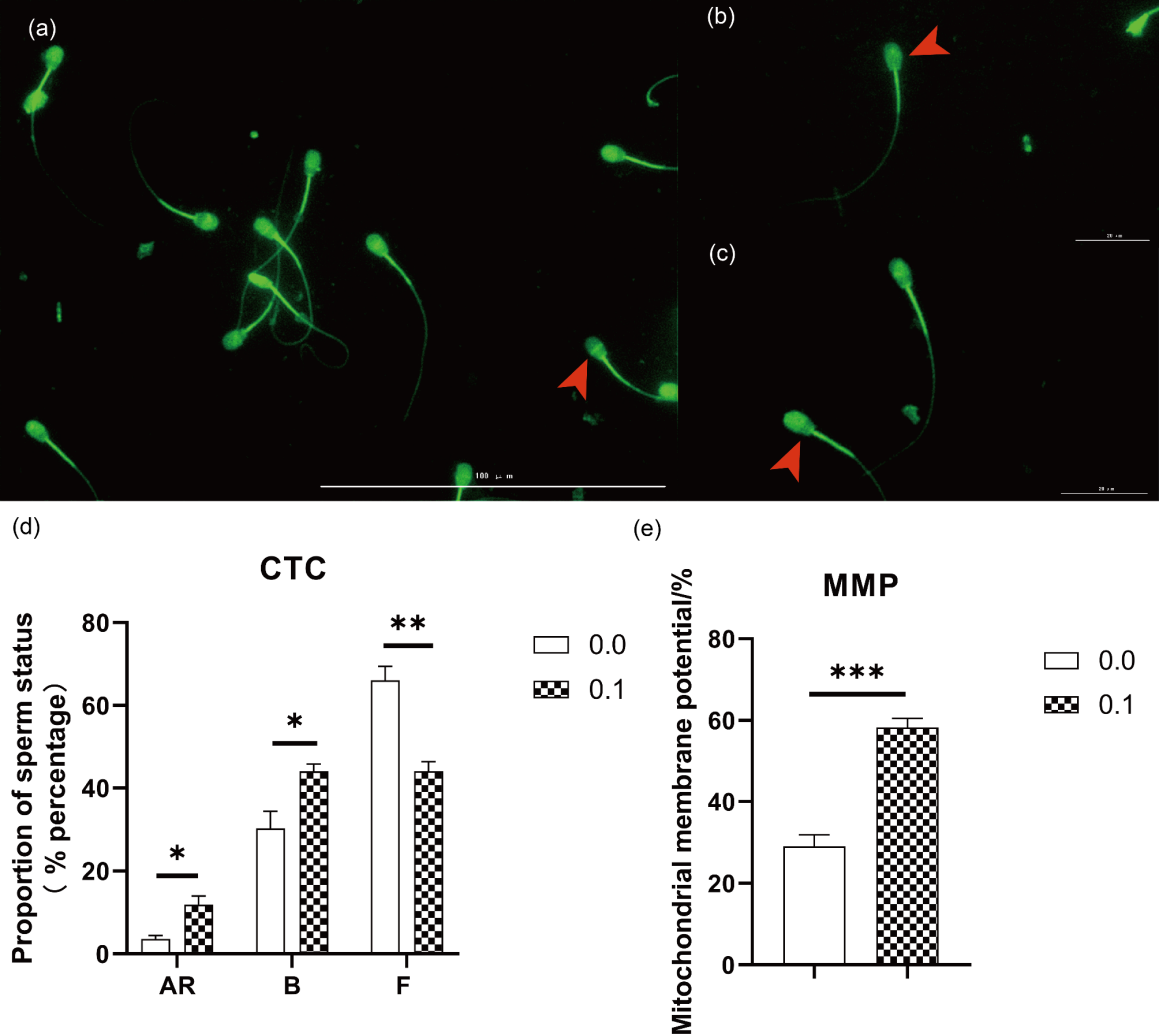
(**a**) Acrosome reacted sperm arising from the CTC fluorescence assay. AR pattern, with dull fluorescence over the whole head except for the thin, bright punctate band of fluorescence in the equatorial segment (arrow). (**b**) Uncapacitated sperm arising from the chlortetracycline fluorescence assay. F pattern, with fluorescence over the whole head (arrow). (**c**) Capacitated sperm arising from the chlortetracycline fluorescence assay. B pattern, with a fluorescence-free band in the post acrosomal region (arrow). (**d**) The effect of PRDX6 on fertility capacity of frozen-thawed sperm. (**e**) Mitochondrial activity of sperm by JC-1 staining. The data were analyzed by t test *P < 0.05, **P < 0.01, *** *P* < 0.001

Three different sperm patterns, such as the AR (acrosome reacted sperm with dull fluorescence over the whole head except for the thin, bright punctate band of fluorescence in the equatorial segment) (Fig. 6a), F (non-capacitated sperm with green fluorescence on the entire head) (Fig. 6b) and B (capacitated sperm with green fluorescence on the acrosomal region and a dark post-acrosomal region) (Fig. 6c) were observed. When compared with the blank control, the percentages of capacitated and acrosome-reacted sperms in the 0.1 mg/L PRDX6 group were increased (*P* < 0.05), while the percentage of non-capacitated sperms was decreased (*P* < 0.01) (Fig. 6d). The MMP of frozen-thawed sperms in the 0.1 mg/L PRDX6 group was higher than in the blank control (Fig. 6e) (*P* < 0.001).

## Discussion

In previous studies, the different protein profiles between GFE and PFE sperms were usually revealed by using the 2D gel-based proteomic method, which has lower sensitivity and worse reproducibility than iTRAQ proteomics. In order to gain more insights into the protein profiles between GFE and PFE buffalo bull sperms, the iTRAQ-coupled LC-MS/MS method was used in this study. 2652 quantified proteins were identified, among which 248 proteins were differentially expressed and many of the DEPs participated in OXPHOS and purine metabolism.

PRDX6, which upregulated in GFE sperm, possessed three enzymatic activities, and located at the position that join OXPHOS with purine metabolism. PRDX6 interacts with multiple redox signaling pathways to interfere with cell proliferation and apoptosis [18]. Our result indicated PRDX6 and OXPHOS were negative correlation, which was somewhat different with the previous knowledge of sperm metabolism. In order to understand the function of PRDX6 in sperm freezability, recombinant PRDX6 protein was added to semen freezing extender during the cryopreservation of Mediterranean buffalo sperm. We found that compared with blank control, the motility and sperm function of frozen-thawed sperm were significantly increased, while the oxidation level was significantly decreased when 0.1 mg/L PRDX6 added. In this study, we report on different freezability spermatozoa with different metabolic preferences. To the best of our knowledge, it is the first report on screening candidate protein related to freezability by iTRAQ proteomics and targeted improving freezer extender for buffalo.

OXPHOS is the process that occurs within the mitochondria and glycolysis occurs in principal part of the sperm [19–21], and is more efficient in terms of ATP production, but it happens at slower rate than glycolysis [22]. Bull sperms rely on both glycolysis and OXPHOS for ATP production, which is different from other species which only rely on one of these processes [23]. However, the abundance of OXPHOS-associated proteins, such as the mitochondrial dehydrogenase family and cytochrome *c* oxidase subunits were significantly decreased in GFE compared with PFE sperm. The KEGG pathway analysis revealed downregulated proteins (GFE vs. PFE) were enriched in the OXPHOS pathway. This indicated that the freezability of buffalo sperm was negatively related to OXPHOS. During the process of OXPHOS, ROS are generated in the electron transport chain [24, 25]. These can induce DNA damage when they are present at high levels which can lead to apoptosis [25]. This may be one of the reasons for the OXPHOS decrease observed in GFE sperm.

Mitochondrial pyruvate carrier 2 (MPC2), is the oligomeric complex component of MPC, and it plays a vital role in transporting pyruvate from the cytosol into organelles [26] and promotes efficient pyruvate transport into proteoliposomes [27]. Pyruvate, the end product of glycolysis, is a key metabolic molecule enabling mitochondrial ATP synthesis and it takes part in multiple biosynthetic pathways within mitochondria [26]. MPC2 and MPC1 form a hetero-complex in the inner mitochondrial membrane and link glycolysis to OXPHOS [28]. The downregulation of MPC2 promotes glycolysis via the mTOR pathway in colorectal cancer cells [28]. Phosphoglycerate mutase (PGAM) is an essential glycolytic enzyme for the spermatozoa energy supply [29]. PGAM2 is the key determinant to maintaining sperm motility and morphology because it is associated to the fibrous sheath of the main segment within the sperm flagellum [30]. The overexpression of PGAM2 interferes with mitochondrial function and this can affect fatty acid metabolism [31]. In this study, the abundance of MPC2 and PGAM2 in GFE sperm showed 0.37-fold decrease and 1.59-fold increase when compared to PFE sperm, respectively. This indicates that the metabolic pattern in GFE sperm might rely on glycolysis, which has the fast rate of ATP generation.

Supervillin is an actin associated protein, which could interact with myosin II, F-actin and cortactin to regulate actin dynamics, finally promote cell contractility and motility [32]. It also decreases the expression of tumor suppressor protein p53 and downstream target genes to increase cell survival [33]. As a cysteine protease inhibitor, cystatin C could preserve sperm fertilizing ability before it reaches the fallopian tube by preventing precocious capacitation and acrosome reaction [34], moreover, it prevents DNA strand breaks and inhibits cathapsin C-mediated cathapsin G leakage to control apoptosis [35]. In this study, the abundance of supervillin and cystatin C in GFE sperm showed 2.00-fold and 1.70-fold increases when compared to PFE sperm, respectively, which indicate that the two proteins might enhance the survival of frozen-thawed sperm by inhibiting apoptosis.

PRDX6 possesses three enzymatic activities, including glutathione peroxidase, acidic calcium-independent phospholipase A_2_ (aiPLA_2_) and lysophosphatidylcholine acyl transferase (LPCAT) activities [36]. These are involved in the regulation of glucose-lipid [37], phospholipid [38] and fatty acid [39] metabolisms. PRDX6 is an important link in the chain of elements connecting redox homeostasis and proliferation [40]. We found that the motility of frozen-thawed sperms was significantly higher upon addition of 0.01, 0.1 and 1 mg/L recombinant PRDX6 when compared to control. ROS and MDA levels were significantly decreased when 0.1 mg/L PRDX6 was added, the T-AOC and 8-OHdG levels had no differences, which may be related to the concentration added. These results suggested that PRDX6 could improve the quality of frozen-thawed sperms by the effect on its peroxidase activity.

Mitochondria is especially sensitive to cryo-damage [21, 41]. Some sperm apoptotic-like changes, such as the MMP loss, are known to be accelerated by cryopreservation [21, 42]. Mitochondrial functionality and an intact MMP are the pre-requisite for sperm motility, hyperactivation, capacitation, acrosin activity, acrosome reaction and DNA integrity [43]. An assessment of these parameters is more effective in assessing of sperm kinetics than CASA. The MMP level of the 0.1 mg/L PRDX6 group was significantly higher than blank control, which illustrated that PRDX6 had a protective effect on mitochondrial function of frozen-thawed sperms.

Sperm capacitation and acrosome reaction are important indicators for measuring the sperm fertilization capacity. Sperm capacitation induces hyperactivation whereby they acquire the ability to undergo the acrosome reaction, which is one of the two post-testicular sperm maturation supporting acquisitions of sperm-fertilizing capacity [44]. This study found that the percentages of capacitation and acrosome reaction in sperm were significantly higher in the 0.1 mg/L PRDX6 group when compared to blank control. This suggested that PRDX6 could improve the frozen-thawed sperms fertilization capacity of Mediterranean buffalo bulls. The changes of the membrane state at low temperatures can cause dysfunction of cholesterol efflux, which may be one of the reasons for the reduced fertility of frozen-thawed sperms [45]. LPCAT is one of the major players in the phosphatidylcholine remodeling pathway and it plays an important role in maintaining cell membrane structure and function [26]. PRDX6 possesses LPCAT activity and this may be another reason for its ability to improve sperm fertilization capacity.

## Conclusions

The metabolic pattern of freezability of spermatozoa obtained from Mediterranean buffaloes was negatively associated with OXPHOS. The addition of PRDX6 to the semen freezing extender provided protective effect on frozen-thawed sperms as judged by its enzymatic activities of LPCAT and peroxidase.

## Materials and methods

### Animals and study design

Eighteen 3 to 5 years old bulls with an average weight of about 770 kg were selected based on the motility of their post-thawed sperms (≥ 30 or < 30%) obtained from 2251 ejaculates from 2018 to 2019. The bulls were raised under the same feeding and management protocols at the Livestock of Poultry Breeding Station of Guangxi Zhuang Autonomous Region in China.

Semen samples were collected using an artificial vagina twice weekly. The quality parameters of fresh semen, such as volume, concentration, motility and kinetics were determined as previous study [46]. The motility was evaluated by 200 phase-contrast microscopy (Nikon, Model Eclipse 80i, Tokyo, Japan) with 100 ×. Kinetic parameters were analyzed under 8 random fields (100 ×) using the CASA system on constant temperature microscope stage at 38.5 °C, which was the SETUP specific for buffalo semen [47]. The settings of CASA system were as follows: temperature = 38.5 °C, frame rate = 60 Hz, frames acquired = 30, minimum contrast = 35, minimum cell size = 5 pixels, cell size = 9 pixels, cell intensity = 110 pixels, progressive cells (VAP cut-off = 50 mm/s, STR cut-off = 70%), slow cells (VAP cut-off = 30 m/s and VSL cut-off = 15 m/s). Ejaculates with general motility percentage of ≥ 60% [46] were used in the study.

For freezing semen, the ejaculates were diluted in BIOXcell™ extender according to the manufacturer’s instructions (IMV, L’Aigle, France). Semen was packaged in 0.25 mL straws and processed for cryopreservation following the standard protocol (lowering of temperature from 4 °C to − 10 °C at 5 °C/min, − 10 °C to − 100 °C at 40 °C/min, and − 100 to − 140 °C at 20 °C/min) in an automatic programmable biological cell freezer (IMV, L’Aigle, France). Then the straws were plunged into liquid nitrogen (− 196 °C) for storage until they were analyzed. Thawing was achieved by immersing the straws in a water bath at 38.5 °C for 30 s.

### iTRAQ-based proteomics analysis

Spermatozoa were separated from the seminal plasma by centrifugation at 4 °C for 20 min at 3000 g, then washed with phosphate buffered saline (PBS) three times and stored at -80 °C until analysis. The sperm protein extraction, trypsin digestion, iTRAQ labeling and mass spectrometry procedures were performed as previously described [15]. Tryptic peptides were analyzed by LC-MS/MS on the Q Exactive HF mass spectrometer coupled with a NanoLC-1000 HPLC system (Thermo, USA). The obtained MS data were compared against the Bos Taurus sequences (37,512 entries) in the uniprot database (2020_07) by using the SEQUEST software integrated into Proteome Discoverer (version 1.3, Thermo Scientific, RRID: SCR_014477). Trypsin was chosen as enzyme and two missed cleavages were allowed. The searches used the peptide mass tolerance of 20 ppm and product ion tolerance of 0.05 Da, which resulted in 5% false discovery rate (FDR) of peptides.

A heatmap and cluster dendrogram of the significant genes were plotted by using R programming language (https://www.r-project.org/, RRID: SCR_001905). When performing hierarchical clustering the Euclidean distance algorithm for similarity measure and the average linkage clustering algorithm (clustering uses the centroids of the observations) for clustering were selected.

Gene Ontology (GO) and Kyoto Encyclopedia of Genes and Genomes (KEGG) enrichment analysis were performed by using the DAVID software (https://david.ncifcrf.gov/, RRID: SCR_001881). KEGG database was used to analyze pathway ( www.kegg.jp/kegg/kegg1.html) [48]. Fisher’s exact test (P-value < 0.05) was used to compare the distribution of GO annotation and KEGG pathways for target protein and overall protein collections. The Clusters of Orthologous Groups of proteins (COG/KOG) (https://www.ncbi.nlm.nih.gov/COG/) database was used to annotate the protein function and pathway. In order to investigate the interactions among various proteins and their involvement in diverse interacting pathways, the protein interaction network (PPI) was analyzed with all the significantly DEPs. The Search Tool for the Retrieval of Interacting Genes/Proteins (STRING) database (http://string-db.org/, RRID: SCR_005223) and Cytoscape software (http://www.cytoscape.org/, RRID: SCR_003032) were also used. The proteome analysis was performed by BIO Science Co (Nanning, China).

### Targeted protein quantification by PRM or WB

The data acquired by the iTRAQ analysis were further verified by quantifying the abundance of 6 selected proteins using PRM. The signature peptides for the target proteins were defined according to the iTRAQ data and unique peptide sequences were selected for the PRM analysis. PRM analysis was carried out on the Q-Exactive HF spectrometer (Thermo, USA). The raw data obtained were analyzed using the Proteome Discoverer and the FDR was set at 1% for peptides. The resulting MS data were processed by using Skyline (version 3.5.0, RRID: SCR_014080).

PRDX6 antibody (Abmart, Cat No T56784, China) was used to quantify the PRDX6 abundance of GFE and PFE sperms, and β-Tublin antibody (Abmart, Cat No M30109, RRID: AB_2916070) was used as control. Goat anti-rabbit mouse IgG-HRP was used as the secondary antibody (Abmart, Cat No M21003, RRID: AB_2920649). Western blot was performed as described previously [13]. Considering the antibodies used were unspecific for buffalo, we cut the membrane prior to hybridization to remove the nonspecific blots. Quantification employed ImageJ (NIH Image J system, USA, RRID: SCR_003070) and the data were normalized to β-Tublin. Each loading sample for WB analysis consisted of three mixed ejaculates.

### Cryopreservation preparation

Three bulls for GFE and PFE groups each were used for the PRDX6 treatment related experiments. Experimental extenders were prepared by the addition of 0, 0.01, 0.1 and 1 mg/L recombinant PRDX6 protein (ABcam, Cat No ab87631, UK), respectively.

### Measurement of oxidative and anti-oxidative levels

For each straw, 200 µL of thawed semen were centrifuged at 1600 × g for 5 min, and the precipitate was collected. The sperm precipitate was mixed with dichlorodihydrofluorescein diacetate (DCFH-DA) (Beyotime, China) medium without fetal bovine serum (FBS) to make the 10 µM DCFH-DA working solution for ROS detection. Samples were protected from light and incubated at 37 °C for 20 min. Spermatozoa were washed gently three times with PBS and the OD was measured at 525 nm with the microplate reader (TECAN, Switzerland).

The spermatozoa were mixed with 400 µL of distilled water and then centrifuged repeatedly to destroy the sperm structure. Finally, they were centrifuged at 4000 × g for 30 min and the supernatants were collected which was regarded as the enzyme crude extract. Lipid peroxidation, oxidative DNA damage and total antioxidant capacity (T-AOC) in the enzyme crude extracts were estimated by using the MDA assay kit (A003-1, Jiancheng, China), 8-OHdG (8-hydroxydeoxyguanosine) ELISA kit (E-EL-0028c, Elabscience, China) and the T-AOC assay kit (A015, Jiancheng, China), respectively. The processes were performed according to the manufacturers’ instructions. The MDA, 8-OHdG and T-AOC levels were assessed by using thiobarbituric acid (TBA), Fe^3+^ reduction and horseradish peroxidase-labeled avidin methods, and measured at 532, 520 and 450 nm on a microplate reader, respectively.

### Sperm functional analyses

Frozen-thawed semen samples were purified in fertilization medium containing heparin (Sigma, USA) by the ‘swim-up’ method for 30 min. Capacitated sperms were collected and stimulated with 20 µM calcium ionophore A23187 (Sigma, USA) for 1 h. The processes of sperm capacitation and acrosome reaction were assessed using the chlortetracycline (CTC) fluorescence assay method according to previous report for bull sperm [49]. MMP (ΔΨm) was measured by using the fluorescent dye, JC-1 (Beyotime, China), as described previously [50]. Sperm images were acquired on the same day using Biotek Cytation5 (Biotek, USA) and an EVOS FL fluorescence microscope (Thermo, USA). Two hundred sperms were counted for each sample.

### Statistical analysis

The two-step hierarchical cluster analysis was performed to classify the freezability of the bulls. All experiments were performed using three independent biological replicates. Aside for the proteomics, all other experiments were repeated three times and statistical significance was tested using the t test and variance (ANOVA) analyses of the SPSS software (version 18.0, RRID: SCR_002865). Data were expressed as mean ± standard error of the mean (SEM). With Pathway Studio, Fisher’s Exact Test was used to determine if the pathways were statistically correlated with DEPs. GraphPad Prism software (version 6.0, GraphPad Software, Inc., USA) was used to draw the graphs. A *P*-value less than 0.05, 0.01 or 0.001 was considered significant differences.

## Electronic supplementary material

Below is the link to the electronic supplementary material.

Supplementary Material 1

Supplementary Material 2

## Data Availability

The mass spectrometry proteomics data of iTRAQ have been deposited to the ProteomeXchange Consortium (http://proteomecentral.proteomexchange.org) via the iProX partner repository with the dataset identifier PXD034388.
