# Supplementary material for: iTRAQ-based comparative proteomics reveal an enhancing role of PRDX6 in the freezability of Mediterranean buffalo sperm
Source: BMC Genomics. 2023 May 5;24:245. doi: 10.1186/s12864-023-09329-x (PMC10163707; doi:10.1186/s12864-023-09329-x)
Supplement: Supplementary file 2 — Supplementary Material 2 [file 12864_2023_9329_MOESM2_ESM.docx]

**Supplementary Figures**

Supplementary Figure 1: The stain free gel of GFE and PFE sperm proteins.


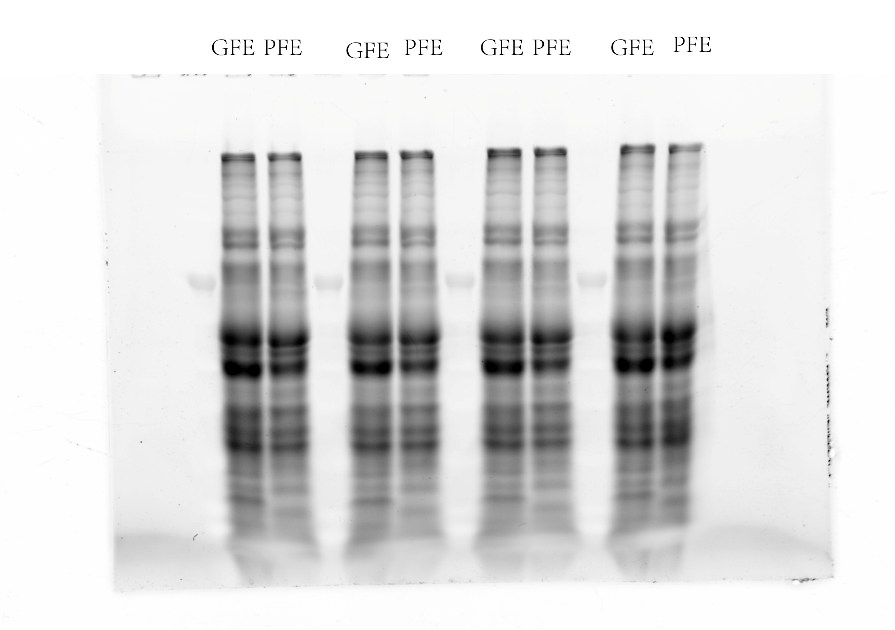


Supplementary Figure 2: The first and second repeats of PRDX6 WB image of GFE and PFE sperm.


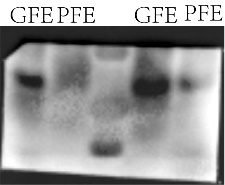


Supplementary Figure 3: The third repeat of PRDX6 WB image of GFE and PFE sperm.


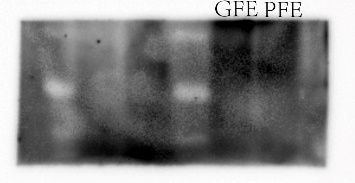


Supplementary Figure 4: The first and second repeats of Tublin WB image of GFE and PFE sperm.


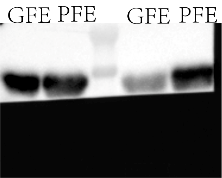


Supplementary Figure 5: The third repeats of Tublin WB image of GFE and PFE sperm.


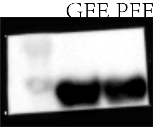


Supplementary Figure 6: MMP was assessed using JC-1 fluorescence dye.


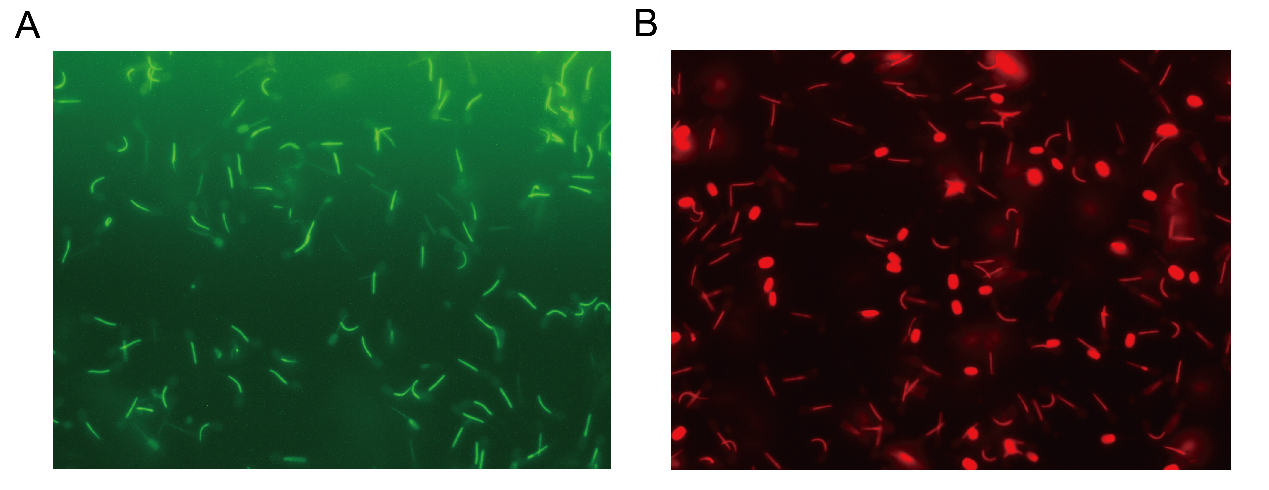


1. The green fluorescence reveals low MMP. (B) The red fluorescence reveals high MMP.
